# The challenges of real-world implementation of web-based shared care software: the HopSCOTCH Shared-Care Obesity Trial in Children

**DOI:** 10.1186/1472-6947-14-61

**Published:** 2014-07-24

**Authors:** Kate Lycett, Gary Wittert, Jane Gunn, Cathy Hutton, Susan A Clifford, Melissa Wake

**Affiliations:** 1 Community Health Service Research, Murdoch Childrens Research Institute, Parkville, VIC, Australia; 2 The University of Melbourne, Parkville, VIC, Australia; 3 Discipline of Medicine, University of Adelaide, Adelaide, SA, Australia; 4 The Royal Children’s Hospital, Parkville, VIC, Australia

**Keywords:** Software, Patient care team, Electronic health records, Pediatric obesity, Primary health care, Tertiary healthcare

## Abstract

**Background:**

E-health initiatives hold promise to improve shared-care models of health care. In 2008–2011 we developed and trialled web-based software to facilitate a randomised trial of a shared-care approach for childhood obesity involving General Practitioners (GPs) working with tertiary specialists. We describe the software’s development, implementation and evaluation, and make recommendations for future e-health initiatives. The web-based software was designed with the goals of allowing both GPs and specialists to communicate and review patient progress; integrating with existing GP software; and supporting GPs to deliver the structured intervention. Specifically, we aimed to highlight the challenges inherent in this process, and report on the extent to which the software ultimately met its implementation and user aims.

**Methods:**

The study was conducted at the Royal Children’s Hospital and 22 general practices across Melbourne, Australia. Participants comprised 30 GPs delivering the shared-care intervention. Outcomes included the following. (1) *GPs’ pre-specified software requirements:* transcribed from two focus groups and analysed for themes using content analysis. (2) *Software implementation and performance* based on the experience of the research team and GPs. (3) *GP users’ evaluation* collected via questionnaire. (4) *Software usage* collected via GP questionnaire and qualified through visual inspection of the software meta-data.

**Results:**

Software implementation posed difficult and at times disabling technological barriers (e.g. out-dated hardware, poor internet connections). The software’s speed and inability to seamlessly link with day-to-day software was a source of considerable frustration. Overall, GPs rated software usability as poor, although most (68%) felt that the structure and functionality of the software was useful. Recommendations for future e-health initiatives include thorough scoping of IT systems and server speed, testing across diverse environments, automated pre-requisite checks and upgrades of processors/memory where necessary, and user-created usernames and passwords.

**Conclusions:**

GPs are willing to embrace novel technologies to support their practice. However, implementation remains challenging mainly for technical reasons, and this precludes further evaluation of potential user-specific barriers. These findings could inform future e-health ventures into shared-care, and highlight the need for an appropriate infrastructure.

**Trial registration:**

Australian New Zealand Clinical Trials Registry: ACTRN126080000553.

## Background

E-health has dramatically transformed the health care sector and is now considered an integral part of health care reform [1]. This is particularly true for primary care (i.e. general practice in Australia), which transitioned rapidly to electronic prescribing and computerised medical records in the early 2000s. However, even for these early adopters, information technology (IT) is yet to include seamless incorporation of decision support, sophisticated patient registers [2], and the sharing and integration of clinical information between hospital-based clinics dealing with chronic conditions and general practice. As complex chronic conditions such as obesity reach record highs, there is increasing interest in IT to enhance the management of these conditions by providing a combination of clinical information, prompts for care, education and improving communication between health care professionals and patients [2,3]. Here, we report our experience in developing, implementing and evaluating web-based software to facilitate a shared-care model of childhood obesity management within the context of an Australian randomised controlled trial in the primary and tertiary care settings.

Globally, overweight and obesity are estimated to affect 10% of children aged 5–17 years. Yet in developed regions, such as the United Kingdom (UK), the Americas and Australia, rates are much higher and are estimated between 25-32% [4-6]. Effective treatment is urgently required if the consequences for these children’s adult health, such as heart disease and premature death, are to be reduced [7]. Specialist obesity clinics providing a multidisciplinary approach have reproducibly documented small improvements in the body composition and health of obese children using lifestyle approaches, sustained for at least 12 months [8,9]. Yet such clinics are inaccessible to most children and have long waiting lists [10]. General practice is the obvious setting to intervene, as it is accessible to most overweight children [11]. General practitioners (GPs) consider that management of childhood obesity falls within their role, but most do not feel that they have the requisite skills or support to manage it [12]. Shared-care programs could offer the specialist skills required to manage childhood obesity within the primary care setting.

Shared-care has been widely implemented for conditions as diverse as childhood cancer [13], adult obesity [14] and antenatal maternity care. However, Cochrane reviews of shared-care programmes effectiveness to manage chronic conditions are mixed [15,16]. These conflicting findings may reflect the challenges of operationalising shared-care, such as ineffective communication between health care professionals. Web-based software supporting shared-care could offer a major advance, with at least one trial already reporting positive results [17]. Evidence suggests that using such tools supports better integrative patient care [18]. If these software tools are to support shared-care, they would need to be integrated into existing software and ensure ready access, accurate record keeping and best use of time [19].

In 2008–11 we conducted a randomised controlled trial [20] testing a model of obesity management in 3–10 year olds shared between GPs and hospital-based obesity specialists. To facilitate this, we developed a web-based shared-care software with the goals of: 1) allowing the obesity specialists and GPs to collaborate and communicate closely in the care of their patients; 2) providing a structured yet efficient approach to weight management care; 3) providing a mechanism to allow both GPs and specialists to record and track patient progress simultaneously; and 4) integrating this with GPs’ existing desktop software.

The trial was successfully conducted [21]. However, the software implementation faced many hurdles that are likely to be encountered in future clinical, as well as research, contexts. We describe the development, implementation and evaluation of this software, as well as the experience of GPs using the system, and make recommendations for similar e-health initiatives. Given the likely increased adoption of shared-care to improve patient care and decrease strain on tertiary care services, learnings from our trial could inform strategies for the optimal use of electronic resources to facilitate shared-care approaches.

## Methods

### Study design and setting

HopSCOTCH (the Shared-Care Obesity Trial in Children, 2008–11, ACTRN12608000055303) was a randomised controlled trial of obesity management for 3–10 year olds delivered by obesity specialists and GPs. It was conducted at The Royal Children’s Hospital and 22 general practices in metropolitan Melbourne (population 4.0 million), Australia. All intervention children attended a tertiary appointment with a paediatrician and dietician specialising in childhood obesity, followed by up to 11 (mean 3.5 (SD 2.5)) general practice consultations over the following year, supported by shared-care web-based software. Details of the HopSCOTCH methods [20] and outcomes [21] are published; briefly, despite high uptake/retention and positive evaluations, the shared-care intervention did not lead to better body mass index (BMI) outcomes over and above usual care [21]. Methods for GP participation and the shared-care software development are detailed below.

The HopSCOTCH trial was approved by the Royal Children’s Hospital Ethics in Human Research Committee (HREC 280178) and the University of Melbourne Human Research Ethics Committee (0827435).

### General practitioners

**Figure 1:**
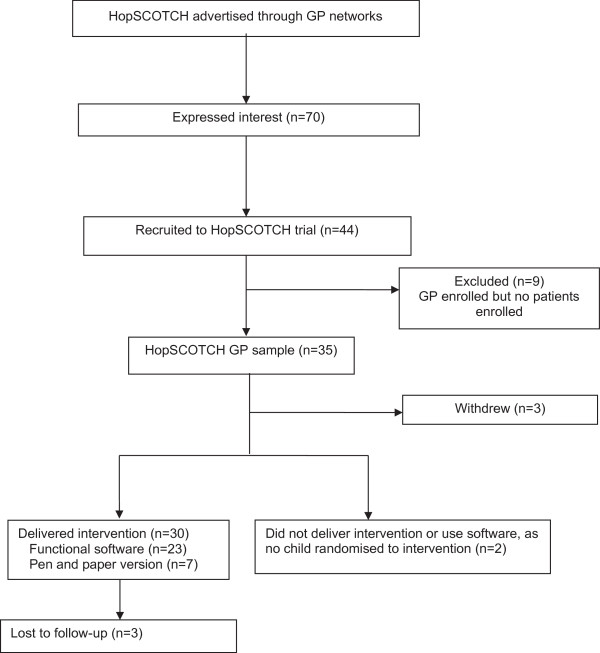
GP participant flow for the HopSCOTCH randomised controlled trial.

GPs were recruited through advertisements as well as personalised invitations sent to those who had participated in our previous primary care paediatric obesity management trials [22,23]. Of the 70 GPs who expressed interest, 35 participated across 22 practices and 30 delivered the intervention (Figure 1).

### Software development

The software was developed in the first year of the project with the expertise of a highly skilled IT consultant and considerable input from GW, CH, JG and the desired users, (i.e. GPs and the specialist obesity clinicians). The obesity specialists were heavily involved in the development of the software, with three specialists forming part of the research team and attending fortnightly meetings throughout the project. We conducted two focus groups, each comprising six interested GPs who were each paid $200 Australian dollars (AUD) for their time. The first session was held prior to commencing software development and GPs were drawn from the Victorian Practice Based Research Network (VicReN), a network for GPs interested in research participation. The second session included only HopSCOTCH GPs and took place when a prototype could be demonstrated.

#### Initial software platform

We initially identified an existing software package for managing adult obesity in general practice (OBEMAN®) [24] as a potential platform for the shared-care software. OBEMAN® had many of the features HopSCOTCH required (i.e. consultation guide, weight management plan, and tracking of anthropometry); however, following feedback from the VicReN focus group, it was not pursued because it lacked some of the key functionality HopSCOTCH required (i.e. linking with desktop software to fit with GPs’ current workflow, web enabled and child focused).

We therefore entered into a partnership with Pen Computer Systems Pty Ltd (PCS), a software company focusing on health care initiatives. PCS had already designed the PrimaryCare Sidebar® to assist in chronic disease management and to support shared-care by allowing multiple clinicians to view and input into a patient’s care at multiple sites in real-time. In collaboration with PCS and our external IT consultant, we developed a new application designed to deliver the intervention, referred to below as the HopSCOTCH software. Software development and quality assurance testing took place over a one year period and involved a number of feedback cycles between the study team (which included GP researchers CH and JG) and the developer, and incorporated a second focus group with GPs recruited into HopSCOTCH prior to finalisation of the software platform.

#### Final software platform

The HopSCOTCH software was embedded within the PrimaryCare Sidebar®. Its key functions were designed around five standardised sequential steps: (1) recording anthropometry; (2) reviewing BMI change, using an online chart to track BMI visually against percentile charts; (3) assessing and tracking progress and motivation; (4) reviewing the care plan (e.g. issues and goals); and (5) providing educational resources. These steps were designed to be repeated at each follow-up visit. For ethical, patient security/confidentiality and financial reasons, the data were stored on a secure server at The Royal Children’s Hospital.

### Data collection procedures

All GPs provided written consent and completed a baseline survey about their practice and demographics. Data regarding desired software functionality were obtained from the GP focus groups, which were recorded and professionally transcribed.

Implementation and usage data were collected by the research team and through inspection of the software meta-data, respectively. Software performance was assessed by intervention GPs’ ‘lived experience’ during the trial as they provided feedback to the research team, as well as a second survey about their experience of the intervention and software at the end of the trial.

### Analysis

The GP focus group transcripts underwent content analysis to identify common themes, which were ranked in order of priority based on the number of times GPs raised the theme. Data obtained through baseline and follow-up GP surveys were summarised using descriptive univariable analyses. GP report of the frequency of appointments and software usage were confirmed by inspecting software meta-data.

## Results

### Characteristics of intervention GPs

GPs (51% male) were mainly aged 45–54 (41%) and 55–64 (33%) years. Half had more than 20 years of experience in general practice and 67% completed their training in Australia/New Zealand.

### GP design considerations

GPs were generally positive about the software design during the focus groups. Of the top ten pre-specified software requirements GPs requested, all but three were achieved (Table 1). Unfortunately, we were not able to fully realise the GPs’ two most important software requirements, i.e. waiting no longer than a few seconds for the software to be launched and being able to work seamlessly within their clinical system and the shared-care software (see Table 1).

**Table 1:** GPs ten highest rated pre-specified requirements identified at the focus group prior to software development

	**GPs top 10 pre-specified software requirements (ranked by priority)**	**Achieved**
1	Speed - waiting longer than a few seconds considered unacceptable	No
2	Ability to work seamlessly within the clinical system (e.g. Medical Director) and shared-care software	No
3	Ability to visually track BMI over time	Yes
4	Information from specialist obesity appointment stored in software	Yes
5	Personalised goals section to allow GP to track motivation and progress	Yes
6	General notes section	Yes
7	Ability to look back and see which goals were achieved	Yes
8	At least three intervention patients per GP so software becomes intuitive	No
9	Ability to keep a record of the information when the study finishes	Yes
10	Communication between GPs and specialists via email	Yes

### Implementation and delivery

To install the software, GPs received an email containing a hyperlink to the download webpage; a prerequisite checklist (e.g. compatibility with operating system, administrator rights); personalised user details, including a username and secure password; and step-by-step installation instructions. Researchers began booking one-on-one software training sessions with GPs and were available by phone to assist with software installation.

Of the 30 GPs, 12 attempted to install the software (Version 1) and only four were successful; two had external IT teams requesting an installation fee, while six encountered error messages or unacceptable download delays. This was largely due to poor internet connection speeds leading to download delays that were unacceptable in a busy family practice environment, or insufficient processing speed/memory. The latter was remedied in the subsequent software release via a pre-requisite processor check. Subsequently, the research team were required to assist with the remaining 26 installations of which several failed. The software took between 15 and 60 minutes (estimated mean 35 minutes) to install. Installation and GP software training was combined into one visit where possible, which often meant training time was reduced.

### Performance

As GPs commenced intervention delivery, they began to contact the research team to: 1) inform that the software was running so slowly it was unusable, and 2) retrieve their user details, despite previously receiving these by email. Subsequent testing revealed that the software was running much slower than expected. Identifying the cause of this sluggish performance was challenging in a live environment, particularly with three IT teams involved - the research team, PCS and The Royal Children’s Hospital (where the server was managed) - so a decision was made to take the software offline for 10 days to complete an upgrade.

The upgrade increased the software’s speed and added new functions, including 1) an automated pre-requisite check to ensure the computer met the minimum software requirements (e.g. memory, processing speed); 2) user access to automatic upgrades; and 3) Windows7 compatibility. The research team reinstalled the upgraded software (Version 2) at all practices and completed a 30-minute one-on-one software training session with those who had not previously received training. GPs received a ‘test’ patient within the software for practise and a two-page quick reference software guide (Additional file 1).

Despite the upgrade, seven GPs remained unable to use the software due to out-dated computers (e.g. Windows 1999, insufficient processing speed or memory) and/or poor internet connections. Hence, a paper version of the software user interface was created to ensure the integrity of the structured intervention was upheld. For each child seen by these GPs, researchers copied the specialist’s care plan to the paper version and faxed it to the GP prior to the child’s first appointment, using a cover sheet and the secure study and practice fax numbers, to ensure patient confidentiality. The paper version was then subsequently faxed between the research team and GP after each appointment.

### Software cost

Of the AUD$640,000 three-year project budget, we initially set aside AUD$101,500 for the software but spent far more. This was despite hosting the software server at The Royal Children’s Hospital without additional costs, as third party quotes to set-up, host and cover maintenance were in the vicinity of AUD$42,424.

### Usage and GP feedback

Of the 30 GPs who delivered the intervention, 27 (90%) completed the follow-up survey, including four of the seven who used the software intermittently and moved to the paper version. GP reported software usage varied greatly; 63% reported that they always used it, 33% sometimes and 4% never. This was confirmed by the software meta-data. Post-hoc analyses revealed no differences in software usage by GP age, gender or the number of intervention patients seen. However, GPs using the paper version reported fewer appointments than software users (on average 2.5 versus 3.5 times).

At the end of the trial GPs were asked to imagine that the software issues (i.e. speed and installation) were addressed and consider whether the software would have a positive impact on the treatment of their patients; 60% reported it did. GPs also rated the ease of using the nine key functions of the software (Table 2). The average rating was ‘difficult’ to ‘neutral’.

**Table 2:** **GP (n=27)**^**1**^**reports of ease of use of the various software functions**

**Software function**	**Didn’t answer (% of GPs)**	**Didn’t use (% of GPs)**	**Ease of use (% of GPs)**			**Ease of use**^**2 **^**Mean (sd)**
			**Difficult/very difficult (0-1)**	**Neutral (2)**	**Easy/very easy (3-4)**	
Opening/login into the Sidebar	4	4	48	7	37	1.8 (1.3)
The ‘speed’ of the Sidebar	4	4	63	7	22	1.1 (1.3)
Sidebar structure (assess➜track progress ➜review plan➜print summary)	4	4	44	15	33	1.8 (1.1)
The BMI tracking chart	4	4	22	22	48	2.3 (1.1)
The ‘Track progress’ tab i.e. recording motivation/progress	4	4	30	15	48	2.1 (1.1)
The ‘Educate’ tab with the tip sheets	4	7	7	33	48	2.5 (0.9)
General useability	7	4	44	26	19	1.5 (1.2)
Switching between practice and Sidebar software	4	7	44	19	26	1.5 (1.3)
Contacting the specialist obesity team via email using the Sidebar	4	44	7	15	30	2.5 (0.9)

^1^including four GPs who ended up using a pen and paper version due to disabling software.

^2^maximum possible score 4.

Most (68%) felt that the software’s structure and functionality helped guide them through intervention consultations, while others provided comments about why it was not helpful (Table 3). Overall, 89% of GPs reported that shared-care was a good approach to manage childhood obesity. GPs also provided general comments about the shared-care approach, many of which revolved around the software (Table 4).

**Table 3:** GP verbatim comments about why the software functionality and structure did not help guide consultations

**Functionality**	The software wouldn’t always open
	Didn’t work
	For 6/12 the Sidebar didn’t work. With Zed Med it needed wider screen to be helpful – otherwise it effectively took over all Zed Med clinical records space. Roll out NBN?
	Speed was a major issue
	When it worked ok
	Program updates as well as changes to my hardware meant I didn’t get a good routine going in using the program but it is a good principle
	Only seemed to work once
	Unfamiliarity with the software meant it proved to be a distraction and I felt I wasn’t fully focussed on the consult/patient
	Used only twice then software stopped working
	The software wouldn’t always open and actually a nuisance and embarrassing
**Structure**	Didn’t seem intuitive to me
	Too ‘clunky’
	I found it quite fiddly and complicated and spent too much time in the consult with the computer rather than talking to the family.
	Used different order in consultation
	Lots of prompts/guides – information already there

GP comments taken verbatim from the follow-up survey.

NBN, National Broadband Network.

**Table 4:** GP verbatim general comments about the shared-care approach for reducing obesity in children and its comorbidities

**Positive**	Excellent program! Very slow & clumsy software. Overall very good – thanks!
	Loved the idea of ready access to specialist in the field & expertise setting goals
	I loved the structured approach that the Sidebar encouraged
	Overall very good program – should be instituted more broadly – so people don’t feel ‘threatened’ by having an overweight child and where they can seek help without feeling ‘labelled’
	I think it is a good model for all aspects of medicine
	Whilst it is difficult at the moment, shared consultations with the obesity team would be ideal. Again roll on NBN!
	It was useful to have contact with the specialists when the progress was poor; it helped me feel confident in continuing the consultation
	I think care focused with GP is much more accessible for patients but GPs problem need more skills training
	I found the training useful and will continue
**Negative**	Overall, it was disappointing only one child was recruited and the software didn’t work – so I can’t really comment on such with limited experience
	My criticisms of the Sidebar are: 1) Need a widescreen, 2) actual program was clumsy, 3) never got the printing to work (didn’t really fuss me), 4) S…L…O…W, interrupted consultations, and 5) worth pursing but definitely need better software

GP comments taken verbatim from the follow-up survey.

NBN, National Broadband Network.

## Discussion

### Principal findings

The software met most but not all the pre-specified requirements for facilitating shared-care; most problematically, it was slow and did not provide the necessary seamless link with the GPs’ desktop software. It was difficult to implement and underperformed in real-world settings. Despite careful design considerations and considerable user input, IT expertise, financial resources and quality assurance testing, we faced difficult and at times disabling software challenges. The shared-care model and structured nature of the intervention reinforced by the software were widely accepted by GPs as positive approaches to manage childhood obesity, but the software implementation and usability issues – many relating to poor-quality existing hardware, software platforms and internet connection in GP practices - detracted from these positive aspects of the trial.

#### Strengths

Of the GPs’ ten highest rated software requirements, more than half were successfully achieved. Supplemented by a parallel paper system for around a quarter of the GPs, the software was able to ensure children received structured weight management over the 12-month period. It is encouraging that just over three quarters of the GPs persisted using the software despite the obvious issues; thus, GPs appear willing to participate in research [12], and to embrace technology in their practice despite infrastructure barriers preventing their full engagement.

#### Limitations

Installation issues may have led GPs to lose confidence in the product before using it, yet the fact that 77% persisted using the software make this unlikely. The software’s sluggish performance and inability to link seamlessly with the GPs’ desktop software were the source of much frustration and interfered with GP workflow. These difficulties took up valuable doctor-patient time, which may have affected the overall therapeutic interaction between the clinician and patient [25]. Out-dated hardware, compounded by poor internet connections, rendered the software unusable for seven GPs. It was beyond the scope of this paper to understand what the perspectives and technology uptake of other stakeholders (particularly childhood obesity specialists, the other main user group) might be. This is because only four obesity specialists used the software, three of whom were part of the research team and had been heavily involved in the design, development and testing of the software.

#### Implications in light of other literature

E-health shows no sign of decline [1,26] despite many e-health projects failing to deliver their promised benefits. For example, the UK recently spent £12.4 billion on *HealthSpace*, an internet-accessible personal electronic health record to allow public patients to manage and access their own information with the rationale that patient involvement in their own care is often beneficial. Despite this massive investment, only 0.13% of the population opened accounts, and users deemed it neither useful or user friendly [27]. A UK initiative to implement national electronic health records in hospitals and specialist community care settings proved similarly overambitious. The implementation process proved extremely complex, and in its early stages showed little benefit for staff or patients, yet support for the initiative remained strong [19,28]. Despite HopSCOTCH’s much smaller scale, GPs echoed many of these sentiments. Implementation was challenging and useability rated as poor, but GPs remained positive about the software’s potential and the overall shared-care approach.

Whether web-based software can enhance shared-care program effectiveness remains unclear. Shared-care models of health care delivery are complex interventions in themselves that vary widely based on definitions and the number of components involved [29,30]. In this context, we attempted an ambitious programme that not only required care providers to change their daily practice and working relationships, but also required novel software to deliver the intervention. Given the complexity involved in such an integrated model, novel software would perhaps in an ideal world be reserved for shared-care models with proven efficacy in order to determine whether software adds benefit. However, this is not always possible – here, for instance, the software was designed to actually help drive the changed daily practice in a way that could not readily be achieved without it – and would also add considerable time to an already-slow research-to-practice cycle. Further, if shared-care models are to become standard practice, standard definitions and adequate evaluation are essential [29,30].

#### Recommendations

Our findings highlight important considerations for future e-health initiatives. Firstly, software development, testing and implementation is an expensive and time consuming endeavour. For those embarking upon such a project we recommend extensive scoping of health professionals’ IT systems (e.g. hardware, software platforms and internet connection speed) prior to software development. Secondly, given the variability in IT systems and infrastructure, thorough quality assurance testing is recommended across diverse IT systems. In addition, we recommend randomising practices into efficacy studies only once the software is working well and that pragmatic effectiveness studies also need to be conducted if the software intervention is found to be efficacious. Installation may be aided by an automated pre-requisite check to ensure the minimum requirements are met, as many practices were not fully aware of the capabilities of their system. An automatic update function is also highly recommended for future software upgrades. Thirdly, we suggest allowing health professionals to choose their own username and password to ensure they remember it. Finally, cloud-based servers now provide an efficient, flexible, scalable and cost-effective solution but the utility of this approach still depends on the speed of connection. In the absence of an appropriate IT infrastructure, web-based shared-care software is perhaps best trialled within an artificial research environment to minimise these ‘real world’ issues.

## Conclusions

The development work required to implement web-based software tools into routine practice cannot be underestimated. Despite engagement of appropriate expertise and considerable investment of money and time in development and testing, fundamental deficiencies in infrastructure proved limiting. GPs appear open to the idea of embracing new technologies to support practice, yet implementing such technologies is hampered by highly variable hardware, operating systems and internet connection speeds. Our experience is not unique. Future research is required to determine whether or not optimized web-based software enhances shared-care programs in real or artificial IT environments.

## Abbreviations

GPs: General practitioners; UK: United Kingdom; IT: Information technology; BMI: Body mass index; PCS: Pen Computer Systems Pty Ltd; VicReN: Victorian Practice Based Research Network; AUD: Australian dollars.

## Competing interests

The authors declare that they have no competing interests.

## Authors’ contributions

KL coordinated the study, conducted the analyses and drafted the manuscript. GW participated in the design of the study and software, and also helped draft the manuscript. JG participated in the design of the study and facilitated the focus groups. CH participated in the design of the study and facilitated the focus groups. SAC helped conduct the analyses and helped draft the manuscript. MW oversaw the project, conceived of the software and helped draft the manuscript. All authors have read and provided critical input into the manuscript and approved the final version.

## Pre-publication history

The pre-publication history for this paper can be accessed here:

http://www.biomedcentral.com/1472-6947/14/61/prepub

## Supplementary Material

Additional file 1 HopSCOTCH software quick reference guide. Click here for file

## References

[B1] Coiera E Why e-health is so hard Med J Aust 2013 198 4 178 179 23451947 10.5694/mja13.10101

[B2] McInnes DK Saltman DC Kidd MR General practitioners’ use of computers for prescribing and electronic health records: results from a national survey Med J Aust 2006 185 2 88 91 16842064 10.5694/j.1326-5377.2006.tb00479.x

[B3] Wan Q Makeham M Zwar NA Petche S Qualitative evaluation of a diabetes electronic decision support tool: views of users BMC Med Inform Decis Mak 2012 12 61 22759239 10.1186/1472-6947-12-61 PMC3426492

[B4] Lobstein T Baur L Uauy R TaskForce IIO Obesity in children and young people: a crisis in public health Obes Rev 2004 5 Suppl 1 4 104 15096099 10.1111/j.1467-789X.2004.00133.x

[B5] Swinburn BA Sacks G Hall KD McPherson K Finegood DT Moodie ML Gortmaker SL The global obesity pandemic: shaped by global drivers and local environments Lancet 2011 378 9793 804 814 21872749 10.1016/S0140-6736(11)60813-1

[B6] Olds T Maher C Zumin S Peneau S Lioret S Castetbon K Bellisle de Wilde J Hohepa M Maddison R Lissner L Sjoberg A Zimmermann M Aeberli I Ogden C Flegal K Summerbell C Evidence that the prevalence of childhood overweight is plateauing: data from nine countries Int J Pediatr Obes 2011 6 5–6 342 360 21838570 10.3109/17477166.2011.605895

[B7] Juonala M Magnussen CG Berenson GS Venn A Burns TL Sabin MA Srinivasan SR Daniels SR Davis PH Chen W Sun C Cheung M Viikari JS Dwyer T Raitakari O Childhood adiposity, adult adiposity, and cardiovascular risk factors N Engl J Med 2011 365 20 1876 1885 22087679 10.1056/NEJMoa1010112

[B8] Hunt LP Ford A Sabin MA Crowne EC Shield JP Clinical measures of adiposity and percentage fat loss: which measure most accurately reflects fat loss and what should we aim for? Arch Dis Child 2007 92 5 399 403 17261578 10.1136/adc.2006.103986 PMC2083742

[B9] Ford AL Hunt LP Cooper A Shield JP What reduction in BMI SDS is required in obese adolescents to improve body composition and cardiometabolic health? Arch Dis Child 2010 95 4 256 261 19966092 10.1136/adc.2009.165340

[B10] Sabin MA Ford A Hunt L Jamal R Crowne EC Shield JP Which factors are associated with a successful outcome in a weight management programme for obese children? J Eval Clin Pract 2007 13 3 364 368 17518800 10.1111/j.1365-2753.2006.00706.x

[B11] Wake M Salmon L Waters E Wright M Hesketh K Parent-reported health status of overweight and obese Australian primary school children: a cross-sectional population survey Int J Obes Relat Metab Disord 2002 26 5 717 724 12032758 10.1038/sj.ijo.0801974

[B12] Gerner B McCallum Z Sheehan J Harris C Wake M Are general practitioners equipped to detect child overweight/obesity? Survey and audit J Paediatr Child Health 2006 42 4 206 211 16630323 10.1111/j.1440-1754.2006.00831.x

[B13] Blaauwbroek R Tuinier W Meyboom-de Jong B Kamps WA Postma A Shared care by paediatric oncologists and family doctors for long-term follow-up of adult childhood cancer survivors: a pilot study Lancet Oncol 2008 9 3 232 238 18282804 10.1016/S1470-2045(08)70034-2

[B14] Richman RM Webster P Salgo AR Mira M Steinbeck KS Caterson ID A shared care approach in obesity management: the general practitioner and a hospital based service Int J Obes Relat Metab Disord 1996 20 5 413 419 8696419

[B15] Gruen RL Weeramanthri TS Knight SE Bailie RS Specialist outreach clinics in primary care and rural hospital settings Cochrane Database Syst Rev 2004 1 CD003798 10.1002/14651858.CD003798.pub2 PMC9016793 14974038

[B16] Smith SM Allwright S O’Dowd T Effectiveness of shared care across the interface between primary and specialty care in chronic disease management Cochrane Database Syst Rev 2007 3 CD004910 10.1002/14651858.CD004910.pub2 17636778

[B17] Blaauwbroek R Barf HA Groenier KH Kremer LC van der Meer K Tissing WJ Postma A Family doctor-driven follow-up for adult childhood cancer survivors supported by a web-based survivor care plan J Cancer Surviv 2012 6 2 163 171 22124938 10.1007/s11764-011-0207-5 PMC3321136

[B18] Featherstone I Keen J Do integrated record systems lead to integrated services? An observational study of a multi-professional system in a diabetes service Int J Med Inform 2012 81 1 45 52 21962435 10.1016/j.ijmedinf.2011.09.002

[B19] Robertson J Moxey AJ Newby DA Gillies MB Williamson M Pearson SA Electronic information and clinical decision support for prescribing: state of play in Australian general practice Fam Pract 2011 28 1 93 101 21109619 10.1093/fampra/cmq031 PMC3023073

[B20] Wake M Lycett K Sabin MA Gunn J Gibbons K Hutton C McCallum Z York E Stringer M Wittert G A shared-care model of obesity treatment for 3–10 year old children: protocol for the HopSCOTCH randomised controlled trial BMC Pediatr 2012 12 39 39 22455381 10.1186/1471-2431-12-39 PMC3464143

[B21] Wake M Lycett K Clifford SA Sabin MA Gunn J Gibbons K Hutton C McCallum Z Arnup SJ Wittert G Shared care obesity management in 3–10 year old children: 12 month outcomes of HopSCOTCH randomised trial BMJ 2013 346 f3092 23751902 10.1136/bmj.f3092 PMC3677741

[B22] Wake M Baur LA Gerner B Gibbons K Gold L Gunn J Levickis P McCallum Z Naughton G Sanci L Ukoumunne OC Outcomes and costs of primary care surveillance and intervention for overweight or obese children: the LEAP 2 randomised controlled trial BMJ 2009 339 b3308 19729418 10.1136/bmj.b3308 PMC2737607

[B23] McCallum Z Wake M Gerner B Baur LA Gibbons K Gold L Gunn J Harris C Naughton G Riess C Sanci L Sheehan J Ukoumunne OC Waters E Outcome data from the LEAP (Live, Eat and Play) trial: a randomized controlled trial of a primary care intervention for childhood overweight/mild obesity Int J Obes (Lond) 2007 31 4 630 636 17160087 10.1038/sj.ijo.0803509

[B24] Abed HS Wittert GA Leong DP Shirazi MG Bahrami B Middeldorp ME Lorimer MF Lau DH Antic NA Brooks AG Abhayaratna WP Kalman JM Sanders P Effect of weight reduction and cardiometabolic risk factor management on symptom burden and severity in patients with atrial fibrillation: a randomized clinical trial JAMA 2013 310 19 2050 60 24240932 10.1001/jama.2013.280521

[B25] Fiks AG Alessandrini EA Forrest CB Khan S Localio AR Gerber A Electronic medical record use in pediatric primary care J Am Med Inform Assoc 2011 18 1 38 44 21134975 10.1136/jamia.2010.004135 PMC3005866

[B26] Fiks AG Designing computerized decision support that works for clinicians and families Curr Probl Pediatr Adolesc Health Care 2011 41 3 60 88 21315295 10.1016/j.cppeds.2010.10.006 PMC3373310

[B27] Greenhalgh T Hinder S Stramer K Bratan T Russell J Adoption, non-adoption, and abandonment of a personal electronic health record: case study of HealthSpace BMJ 2010 341 c5814 21081595 10.1136/bmj.c5814 PMC2982892

[B28] Sheikh A Cornford T Barber N Avery A Takian A Lichtner V Petrakaki D Crowe S Marsden K Robertson A Morrison Z Klecun E Prescott R Quinn C Jani Y Ficociello M Voutsina K Paton J Fernando B Jacklin A Cresswell K Implementation and adoption of nationwide electronic health records in secondary care in England: final qualitative results from prospective national evaluation in “early adopter” hospitals BMJ 2011 343 d6054 22006942 10.1136/bmj.d6054 PMC3195310

[B29] Ouwens M Wollersheim H Hermens R Hulscher M Grol R Integrated care programmes for chronically ill patients: a review of systematic reviews Int J Qual Health Care 2005 17 2 141 146 15665066 10.1093/intqhc/mzi016

[B30] Mitchell GK Tieman JJ Shelby-James TM Multidisciplinary care planning and teamwork in primary care Med J Aust 2008 188 8 Suppl S61 64 18429739 10.5694/j.1326-5377.2008.tb01747.x

