# Supplementary material for: The challenges of real-world implementation of web-based shared care software: the HopSCOTCH Shared-Care Obesity Trial in Children
Source: BMC Med Inform Decis Mak. 2014 Jul 24;14:61. doi: 10.1186/1472-6947-14-61 (PMC4115470; doi:10.1186/1472-6947-14-61)
Supplement: Additional file 1 — HopSCOTCH software quick reference guide. [file 1472-6947-14-61-S1.pdf]

Introduction

The HopSCOTCH software supports a shared-care process for children with weight management problems. This document provides short instructions on the most common activities that you will need to perform with the software.

For more detailed information a HopSCOTCH User Guide is available.

Log in

You will have received your Username and Password from the HopSCOTCH Co-ordinator. To display the HopSCOTCH sidebar, move the mouse pointer to the right-hand side of the screen or click the HopSCOTCH task bar icon.

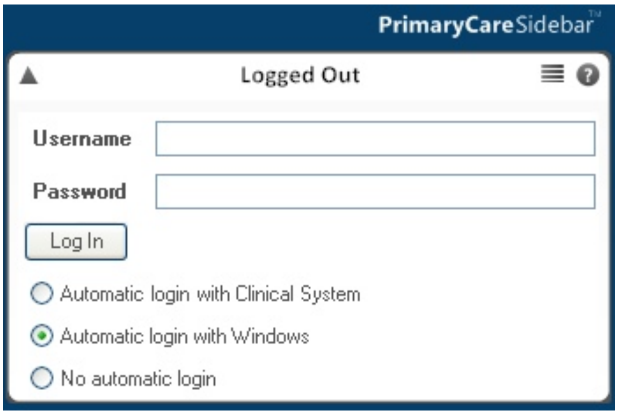

Type your Username and Password and click Log In to access your HopSCOTCH patients.

Selecting a Patient

After log in the software displays a patient list.

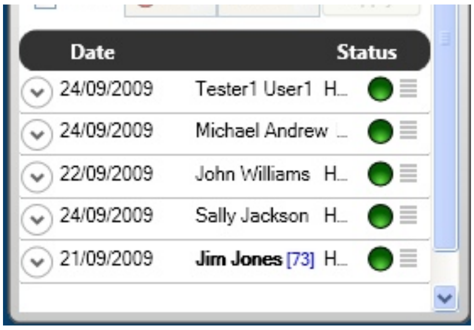

Double click the patient name to display the HopSCOTCH Program window. Click on the menu icon on the right for more actions.

HopSCOTCH Program Window

All the information related to the patient's involvement in the HopSCOTCH program appears here. The window contains four tabs:

Summary	Provides an overview of HopSCOTCH information.
Assess	Entry of assessment measures and a graph of BMI.
Manage	A basic clinical summary, and the patient's goals and actions that are part of the HopSCOTCH management plan.
Educate	Web and downloadable information resources for patients and health care providers.

HopSCOTCH Summary

Click the Summary tab to view a list of recent HopSCOTCH activity and measures, the patients current goals, and the HopSCOTCH care team.

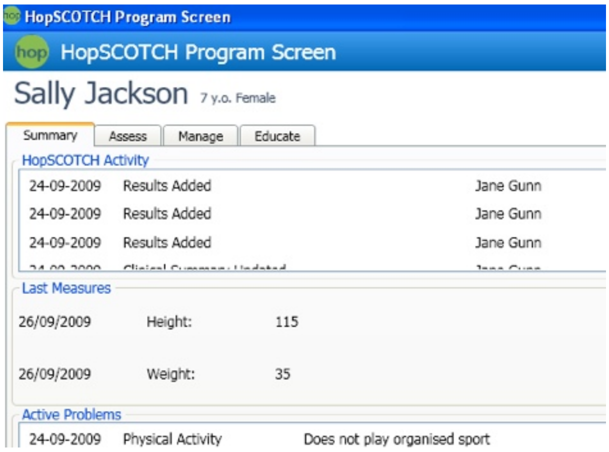

You can print the Summary window by clicking the Print button.

Progress Notes and Attachments

On the Summary tab there exists functionality to enter a Note with an attached file. This helps record more detailed information on the patient's record for sharing with the care team.

Click 'New Note' to display a form to type a description of the attachment. Then you click Attachment to select the file you wish to share.

Assessment Viewing and Entry

At each consultation with a HopSCOTCH patient it is important to record at least the current Height and Weight measures. Click the Assessment tab to view the last measures taken and the plot of BMI history.

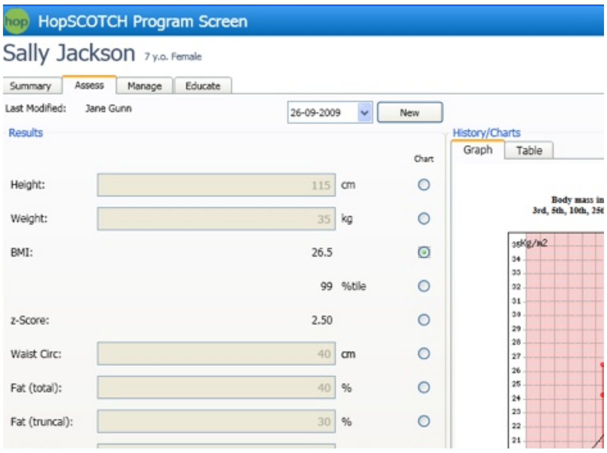

Click the New button to clear the entry boxes for you to type in the measures taken today. You can also type any other Notes from the Assessment. Click Save when you have entered all the measures taken.

You can view charts for other measures by clicking the radio button to the right of the measure value.

Clinical Summary and Active Problems

The first sub-tab under the Manage tab is Active Problems. This tab contains a summary of the patient's clinical condition. This should include the ordering clinician's response to any blood tests that the patient under goes.

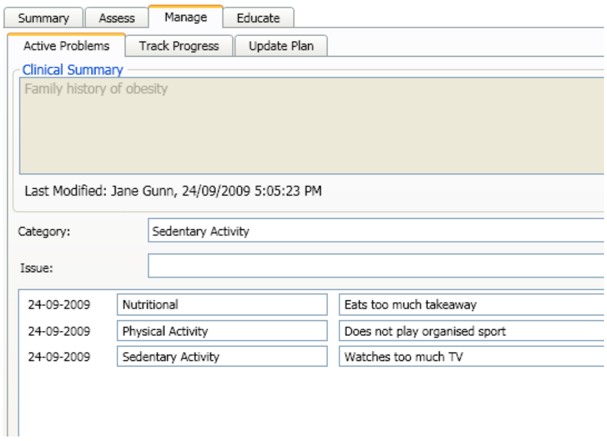

Click Edit to change the Clinical Summary. You can add Active Problems, which are a clinical perspective on a patient's weight management issues, and appear in the Summary print out.

Track Progress

Also important at each HopSCOTCH consultation is tracking the progress of the patient on achievement of the agreed actions.

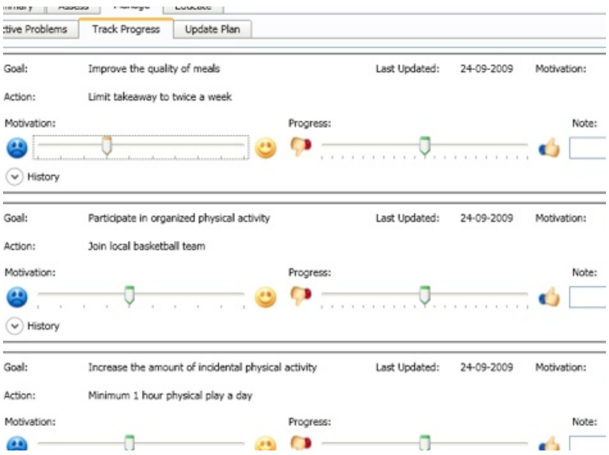

To update the patient's status on a specific action, click in on the Progress slider and drag it to the appropriate value. You can do the same with the Motivation value. After updating these values, click the Update button to store them.

Update Plan

With the final sub-tab on the Manage tab, Update Plan, you set and amend the Goals and Actions agreed with the patient. These Actions are the patient's component of the management plan.

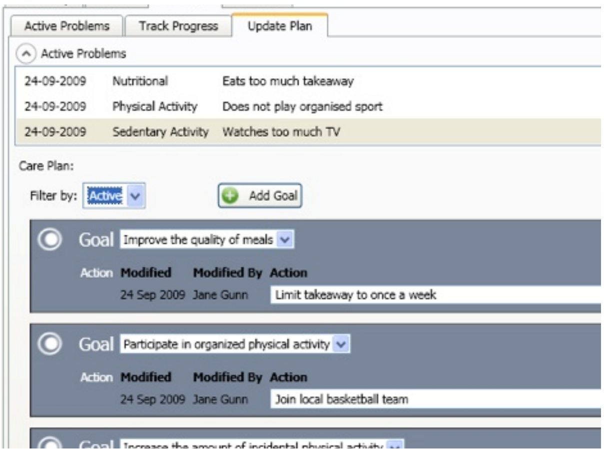

Click on the Action text to edit it. You can also change the Action status. Only 'Active' Actions appear in the Track Progress tab. To add to the patient's Action list, click Add Goal, choose a Goal from the drop down and type the specific action that the patient will take. After completing your update of the Goals and Actions, click Save Care Plan.
